# Utility of the web excision with pre-membranous incision for congenital intestinal atresia-type I and stenosis

**DOI:** 10.1186/s12887-024-04925-6

**Published:** 2024-07-15

**Authors:** Hironori Kudo, Takuro Kazama, Taichi Fukuzawa, Ryo Ando, Ryuji Okubo, Tsuyoshi Sakurai, Masatoshi Hashimoto, Yuki Endo, Masaki Nio, Motoshi Wada

**Affiliations:** Departments of Pediatric Surgery, Tohoku University School of Medicine, Sendai, Japan

**Keywords:** Congenital intestinal atresia, Congenital intestinal stenosis, Web excision, Trasns anastomotic tube

## Abstract

**Purpose:**

This study analyzed the efficacy of web excision combined with a pre-membranous incision on the dilated proximal segment for congenital intestinal atresia with type I and stenosis (CIA-I/S).

**Patients and methods:**

Twenty-six patients underwent surgery for CIA-I/S from January 1990 to June 2022. Patients were categorized into 3 groups according to the surgical procedure: Group A, web excision with pre-membranous incision of the dilated intestine (*n* = 14); Group B, enteroplasty with a trans-membranous vertical incision (*n* = 7) and Group C, diamond-shaped anastomosis (*n* = 5). To minimize the impact of obstruction location on outcomes, we specifically examined 17 cases of duodenal atresia/stenosis: Group D-A, (*n* = 6); Group D-B, (*n* = 6) and Group D-C, (*n* = 5). We retrospectively compared the operative and postoperative parameters among the three groups.

**Results:**

No patient experienced anastomotic leakage or obstruction. There were no significant differences in operative duration or blood loss among the 3 Groups. The median time to feeding initiation was 4, 6.5, and 5 days in Groups A, B, and C, respectively (*p* = 0.04) and was 4, 6.5, and 5 days in Groups D-A, D-B, and D-C, respectively (*p* = 0.04).

**Conclusion:**

Web excision, when compared to enteroplasty and diamond-shaped anastomosis, showed comparable results in terms of the operative duration and postoperative complications. However, it may allow for an earlier initiation of enteral nutrition.

## Introduction

Congenital intestinal atresia or stenosis, including gastric and duodenal atresia/stenosis, is a common cause of neonatal intestinal obstruction. These diseases were classified using Louw’s classification, modified by Grosfeld [1]. In type I congenital intestinal atresia/stenosis (CIA-I/S), the obstruction is caused by an intrinsic membrane, and the dilated proximal bowel and collapsed distal bowel are in continuity with the intact mesentery [2]. Bethell GS, et al. reported that the incidence of congenital duodenal obstruction in the UK was estimated at 1.22 cases per 10 000 live births with the rate of type I being 38% [3]. Furthermore, type I of congenital duodenal atresia, also known as duodenal web, had an estimated incidence of 1 in 10, 000 to 1 in 40,000 live births [4]. The incidence of jejunoileal atresia was approximately 1 to 3 per 10,000 live births, with type I rates reported as 10/43 (23%) and 21/83 (25%) respectively [4–6].

Therapeutic options for type I of congenital duodenal atresia and duodenal stenosis include duodenoduodenostomy or duodenotomy with excision/lysis of the web, and for type I jejunoileal atresia, they are resection with primary anastomosis and web excision [4]. Of these surgical methods, we have adopted web excision as the basic surgical procedure for these diseases, regardless of the location. In the method of web excision, the enteroplasty with a trans-membranous vertical incision was generally performed, which we initially adopted [7–9]. However, in 1998, Sato et al. reported the advantage of web excision with horizontal incision on the oral side of the dilated intestine, which better maintained the natural alignment of the intestinal tract, for the type I of jejunoileal atresia [10]. Since then, we have routinely employed web excision procedure from 2002 onward.

The aim of this study was to analyze the utility of our current procedure, web excision with a pre-membranous incision of the dilated intestine, compared to other procedures that we employed for CIA-I/S from 1990 to 2022.

## Materials and methods

### Patients

One hundred and ten patients underwent surgery for congenital intestinal atresia or stenosis, including gastric and duodenal atresia/stenosis, at our hospital from January 1990 to June 2022. Among them, 30 patients had CIA-I/S. Two patients with multiple atresia and two patients whose postoperative course details were uncertain were excluded from this study. Twenty-six patients with CIA-I/S were then divided into 3 groups according to the surgical procedure: Group A, web excision with a pre-membranous incision of the dilated intestine proximal to the atresia (*n* = 14); Group B, enteroplasty of a trans-membranous vertical incision (*n* = 7); and Group C, diamond-shaped anastomosis (*n* = 5). No significant difference was found among the 3 groups in patient demographic information (Table 1). The trans anastomotic tube (TAT) was placed in 7 patients: 4, 2, and 1 in Groups A, B, and C, respectively. We retrospectively compared several clinical parameters related to the surgical procedure and the postoperative course among the 3 groups.

**Table 1:** Patient demographics (26 patients with CIA-I/S)

Group	A: (14 cases)	B: (7 cases)	C: (5 cases)	*p*
Gestational age (weeks)*	38 (37–41)	38 (36–41)	38 (37–40)	0.93
Female: Male	7:7	3:4	2:3	0.91
Birth weight (g)*	2934 (1990–3424)	2976 (2266–3430)	2602 (2280–3436)	0.99
Prenatal diagnosis	8 (57.1%)	2 (28.6%)	2 (40%)	0.44
Presence of 21 trisomy	2 (14.3%)	2 (28.6%)	2 (40%)	0.46
Age at operation (days)*	1 (1-908)	12.5 (1-495)	8 (0–31)	0.47

Data are presented as the number of cases (%), except for items marked with a *, which are median (range)

CIA-I/S: Congenital intestinal atresia or stenosis in type I and congenital intestinal stenosis

In addition, we focused on 17 patients with duodenal atresia/stenosis to eliminate the effect of different sites of obstruction on outcomes. Similarly, 17 patients were then divided into 3 groups: Group D-A, web excision with a pre-membranous incision of the dilated intestine proximal to the atresia (*n* = 6); Group D-B, enteroplasty of a trans-membranous vertical incision (*n* = 6); and Group D-C, diamond-shaped anastomosis (*n* = 5). TAT was placed in 4 patients: 1, 2, and 1 in Groups D-A, D-B and D-C, respectively. There was no significant difference among the subgroups in patient demographic information (Table 2).

**Table 2:** Patient demographics (17 cases with duodenal lesion only)

Group	D-A: (6 cases)	D-B: (6 cases)	D-C: (5 cases)	*p*
Gestational age (weeks)*	38 (37–39)	38 (36–39)	38 (37–40)	1.0
Female: Male	3:3	3:3	2:3	0.93
Birth weight (g)*	2680 (2426–3424)	2855 (2266–3430)	2602 (2280–3436)	0.54
Prenatal diagnosis	4 (66.7%)	2 (33.3%)	2 (40%)	0.48
Presence of 21 trisomy	2 (33.3%)	2 (33.3%)	2 (40%)	0.97
Age at operation (days)*	2.5 (1-908)	3 (1-495)	8 (0–31)	0.47

Data are presented as the number of cases (%), except for items marked with a *, which are group median (range)

The establishment of postoperative feeding was evaluated using following parameters; the postoperative day (POD) of the initiation of oral, nasogastric tube feeding or TAT feeding (Start of feeding, SF) and the POD of achieving full feeding (> 150 mL/kg/day) by oral, nasogastric tube or TAT (Full Feeding, FF). One patient with colonic atresia who required stoma formation in Group B and another deceased one in Group A were excluded from the analysis of SF and FF.

This study was approved by the ethics committee of our institution (IRB number:26474).

### Surgical procedures

A standard upper abdominal transverse incision was used for the majority of patients in Groups B and C. Since 2008, the circumumbilical omega-shaped incision was used for the majority of patients in Group A, especially in neonates.

**Fig. 1:**
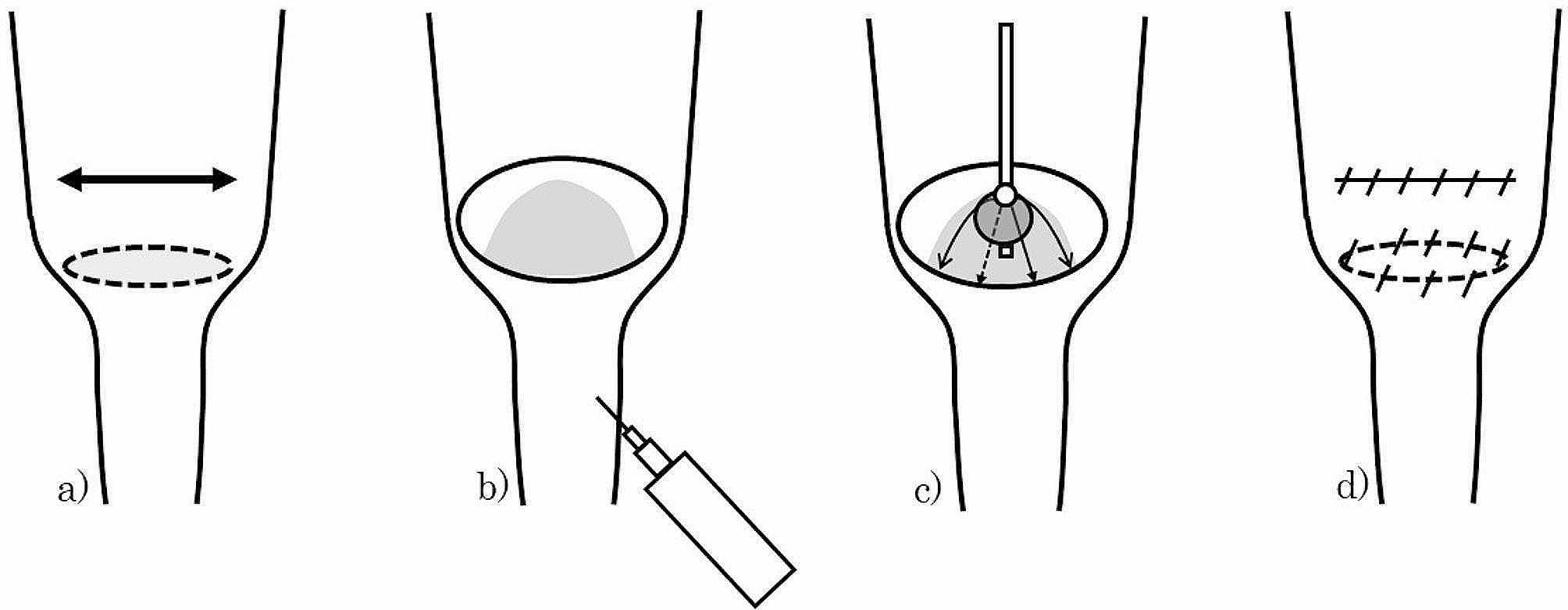
Surgical techniques of web excision with a pre-membranous approach. (**a**) Transverse or longitudinal enterotomy is performed over 1/3 to 1/2 of the bowel circumference, approximately 1 cm above the membrane. (**b**) In cases of atresia, air is introduced into the distal collapsed bowel through a fine needle, to make the web easier to recognize by inflating it with air. (**c**) A balloon-catheter is utilized to delineate the margin of the web. The web is then incised into 4 sections and excised. (**d**) The enterotomy is sutured closed following the same orientation as the incision

Group A: The web excision was performed using a previously described procedure [10]. In brief, the membrane was observed through a serosa in the upper part of caliber change. A transverse enterotomy through 1/3 to 1/2 of the bowel circumference or longitudinal enterotomy was made at 1 cm above the membrane in the oral dilated intestine (Fig. 1a). In the case of atresia, air was pumped into the distal collapsed bowel through a fine needle, to make the web easier to recognize by inflating it with air. (Fig. 1b). The center of the web was incised, the incision was extended in 4 directions, and the web was excised into 4 pieces. After removing the web, we inserted a balloon catheter distally through the base of the removed web, insufflated the balloon, pulled the catheter, and confirmed that no residual web was present. In the case of perforated web, we inserted a balloon catheter through the orifice and incised the web in the same manner, confirming the margin of the web with an insufflated balloon. (Fig. 1c).

Several stitches were placed on the margin of the removed web for hemostasis. The enterotomy was closed in the same direction as the incision line (Fig. 1d).

Groups B and C: The procedures were performed as previously published elsewhere [7, 11].

For TAT placement, the decision to place it or not was made by each surgeon. After having confirmed the passage of the lower intestine, the feeding by TAT was started.

### Statistical analysis

Statistical analyses were performed using the Chi-square test, analysis of variance (ANOVA) or Kruskal-Wallis test firstly. If the result was significant, the significance of differences between groups was determined using Tukey’s *t-*test. A *p-*value of less than 0.05 was considered statistically significant. Statistical analysis was performed using the commercially available software JMP Pro 17.0.0 (SAS Institute Japan Ltd., Tokyo, Japan).

## Results

The site of obstruction in Group A was the duodenum (*n* = 6), jejunum (*n* = 4), ileum (*n* = 3), and pylorus (*n* = 1). In Group B, it was the duodenum (*n* = 6) and the colon (*n* = 1). All 5 patients in Group C had duodenal atresia/stenosis and underwent a diamond-shaped duodenoduodenostomy. The reason for employing this procedure in Group C was that the papilla of Vater could not be recognized or was located on the web.

There was no significant difference in the operative duration and intraoperative blood loss among the 3 Groups (Table 3). None of the patients in either group required additional surgical treatment due to complications such as anastomotic leakage, intestinal obstruction or papillary injury. One patient with ileal atresia of group A died of sepsis unrelated to the surgery 5 days after the web excision. The median Start of feeding Onset (SF) was 4, 6.5, and 5 days in Groups A, B and C, respectively (*p* = 0.049). SF was significantly earlier in Group A than in Group B (*p* = 0.04), while there was no significant difference between Groups A and C (*p* = 0.86). The median Full Feeding (FF) was 12, 12.5, and 11 days in Groups A, B, and C, respectively (*p* = 0.3). In cases without the placement of TAT, the median SF was 4, 7, and 5.5 days in Groups A, B and C, respectively (*p* = 0.02). The median FF was 11, 12.5, and 11 days in Groups A, B, and C, respectively (*p* = 0.3).

**Table 3:** Operative data and postoperative data (26 patients with CIA-I/S)

Group	A: (14 cases)	B: (7 cases)	C: (5 cases)	*p*
Operative durations (minutes)*	150 (102–225)	144 (70–190)	139 (127–235)	0.7
TAT placement	4 (28.6%)	2 (28.6%)	1 (20%)	0.93
Blood loss (ml)*	6.5 (0–42)	6 (0–12)	4 (0–41)	0.63
Start of feeding after operation (days)*	4 (2–7)	6.5 (4–9)	5 (3–6)	0.049
Full feeding after operation (days)*	12 (6–37)	12.5 (9–25)	11 (9–15)	0.3
Start of feeding after operation except for cases using TAT (days)*	4 (3–7)	7 (6–9)	5.5 (3–6)	0.023
Full feeding after operation except for cases using TAT (days)*	11 (7–37)	12.5 (9–16)	11 (9–12)	0.3
Anastomotic leakage	0 (0%)	0 (0%)	0 (0%)	
Anastomotic obstruction	0 (0%)	0 (0%)	0 (0%)	
Death	1 (8.3%)	0 (0%)	0 (0%)	0.75

Data are presented as the number of cases (%), except for items marked with a *, which are the group median (range)

One patient with colonic atresia who required stoma formation in Group B and another deceased one in Group A were excluded from the analysis of feeding onset and full feeding

TAT: Trans-anastomotic tube

All the 5 neonatal patients in Group D-A underwent circumumbilical incision, and most patients in Group D-B and D-C underwent upper abdominal transverse incision. There was no significant difference in the operative duration and intraoperative bleed loss among the 3 groups (Table 4). The median SF was 4, 6.5, and 5 days in Groups D-A, D-B, and D-C, respectively (*p* = 0.04). SF was significantly earlier in Group D-A than in Group D-B (*p* = 0.04), while there was no significant difference between Groups D-A and D-C (*p* = 0.74). The median FF was 11.5, 12.5, and 11 days in Groups D-A, D-B, and D-C, respectively (*p* = 0.38) (Table 4). In cases without the placement of TAT, the median SF was 4, 7, and 5.5 days in Groups D-A, D-B, and D-C, respectively (*p* = 0.04). The median FF was 11, 12.5, and 11 days in Groups D-A, D-B, and D-C, respectively (*p* = 0.56).

**Table 4:** Operative and postoperative data (17 cases with duodenal lesion only)

Group	D-A: (6 cases)	D-B: (6 cases)	D-C: (5 cases)	*p*
Operative durations (minutes)*	169 (124–225)	159.5 (70–190)	139 (127–235)	0.54
TAT placement	1 (%)	2 (33.3%)	1 (20%)	0.77
Blood loss (ml)*	9.5 (0–25)	6.5 (0–12)	4 (0–41)	0.76
Start of feeding after operation (days)*	4 (3–6)	6.5 (4–9)	5 (3–6)	0.04
Full feeding after operation (days)*	11.5 (7–14)	12.5 (9–25)	11 (9–15)	0.38
Start of feeding after operation except for cases using TAT (days)*	4 (3–6)	7 (6–9)	5.5 (3–6)	0.04
Full feeding after operation except for cases using TAT (days)*	11 (7–14)	12.5 (9–16)	11 (9–12)	0.56
Anastomotic leakage	0 (0%)	0 (0%)	0 (0%)	
Anastomotic obstruction	0 (0%)	0 (0%)	0 (0%)	
Death	0 (0%)	0 (0%)	0 (0%)	

Data are presented as the number of cases (%), except for items marked with a *, which are group medians (range)

TAT: Transanastomotic tube

## Discussion

In CIA-I/S, enteroplasty with a trans-membranous vertical incision is commonly employed, and previous research has demonstrated favorable outcomes associated with this procedure [7–9]. In contrast, Sato et al. reported in 1998 two advantages of web excision with a horizontal incision of the dilated intestine proximal to the atresia [10]; one was to maintain the natural alignment of the intestinal tract, and the other was reducing the risk of damage to the intestinal circular muscle by the horizontal incision. Although we initially performed enteroplasty with a trans-membranous vertical incision for CIA-I/S, we agreed with this concept and procedure and have routinely employed it since 2002. In this study, while there was no significant difference among 3 groups in the operative duration and intraoperative blood loss, earlier establishment of enteral nutrition was expected in Group A and D-A compared to Group B and D-B and these results were same in patients without TAT. We think that the smooth alignment of the intestinal tract, reducing the risk of damage to the intestinal circular muscle by the horizontal incision and only removing the mucosa and submucosa without disrupting the integrity of the intestine contributed to favorable tendency in Group A and D-A.

The diamond-shaped duodenoduodenostomy has been reported as the standard procedure for congenital duodenal obstruction, with or without the web [11, 12]. The current study also demonstrated favorable outcomes with this procedure, almost equivalent to those of web excision procedures. However, Duodenoduodenostomy without the excision of the diaphragm is associated with the risk of missing the web, including the windsock abnormality, and performing the anastomosis at the wrong side of the diaphragm. To avoid such a problem, we would add the importance of checking the web of proximal and distal duodenum with a balloon catheter and so on. Other bypass procedures such as duodenojejunostomy are not preferred techniques because they involve a major anastomosis with inherent postoperative problems such as stomal ulceration and blind loop syndrome [8, 13–15].

However, duodenotomy and web excision may result in inadvertent damage to the biliary and pancreatic ducts as the duct may sometime open into the diaphragm [13]. As a transverse enterotomy is made through 1/3 to 1/2 of the bowel circumference at 2 cm above the membrane in the oral dilated intestine for a duodenal lesion, we believe the risk of injury to the papilla of Vater is very low. If the papilla of Vater could not be recognized or was located on the web, at this point, it is also possible to switch to diamond-shaped duodenoduodenostomy. Even if the papilla of Vater is seen, it should not be forgotten that there may be more than one bile duct opening on both sides of the atretic segment in duodenal atresia.

This procedure which Sato et al. employed for jejunoileal atresia [10], was performed for various lesions including the duodenum and the pylorus in this study. These results suggested the high utility of this procedure.

We acknowledged that the current study has some limitations, such as its retrospective study design and the limited number of cases. Therefore, a prospective study with more cases is needed to draw definitive conclusions.

In conclusion, this study demonstrated that the clinical outcomes of CIA-I/S were satisfactory. While web excision was comparable to enteroplasty and diamond-shaped anastomosis in terms of the operative duration and postoperative complications, earlier onset of enteral nutrition might be expected with web excision procedure compared to other procedures.

## Data Availability

The datasets during and/or analysed during the current study available from the corresponding author on reasonable request.
